# Serum circular RNAs act as blood-based biomarkers for hypertrophic obstructive cardiomyopathy

**DOI:** 10.1038/s41598-019-56617-2

**Published:** 2019-12-30

**Authors:** Kristina Sonnenschein, Adriana Luisa Wilczek, David de Gonzalo-Calvo, Angelika Pfanne, Anselm Arthur Derda, Carolin Zwadlo, Udo Bavendiek, Johann Bauersachs, Jan Fiedler, Thomas Thum

**Affiliations:** 1 Institute of Molecular and Translational Therapeutic Strategies (IMTTS), Hannover Medical School, Hannover, Germany; 2 Department of Cardiology and Angiology, Hannover Medical School, Hannover, Germany; 3 Institute of Biomedical Research of Barcelona (IIBB) - Spanish National Research Council (CSIC), 08036 Barcelona, Spain; 4 CIBERCV, Institute of Health Carlos III, 28029 Madrid, Spain; 5 Biomedical Research Institute Sant Pau (IIB Sant Pau), 08025 Barcelona, Spain; 6 National Heart and Lung Institute, Imperial College London, London, UK; 7 REBIRTH Center of Translational Regenerative Medicine, Hannover Medical School, Hannover, Germany

**Keywords:** Biomarkers, Cardiology, Diseases

## Abstract

Hypertrophic cardiomyopathy (HCM) is one of the most common hereditary heart diseases and is associated with a high risk of sudden cardiac death. HCM is characterized by pronounced hypertrophy of cardiomyocytes, fiber disarray and development of fibrosis and can be divided into a non-obstructive (HNCM) and obstructive form (HOCM) therefore requiring personalized therapeutic therapies. In the present study, we investigated the expression patterns of several circulating circular RNAs (circRNAs) as potential biomarkers in patients with HCM. We included 64 patients with HCM and 53 healthy controls to the study and quantitatively measured the expression of a set of circRNAs already known to be associated with cardiac diseases (circDNAJC6) and/or being highly abundant in blood (circTMEM56 and circMBOAT2). Abundancy of circRNAs was then correlated to relevant clinical parameters. Serum expression levels of circRNAs DNAJC6, TMEM56 and MBOAT2 were downregulated in patients with HCM. The inverse association between circRNA levels and HCM remained unchanged even after adjusting for confounding factors. All circRNAs, evaluated separately or in combination, showed a robust discrimination capacity when comparing control subjects with HCM, HNCM or HOCM patients (AUC from 0.722 to 0.949). Two circRNAs, circTMEM56 and circDNAJC6, significantly negatively correlated with echocardiographic parameters for HOCM. Collectively, circulating circRNAs DNAJC6, TMEM56 and MBOAT2 can distinguish between healthy and HCM patients. In addition, circTMEM56 and circDNAJC6 could serve as indicators of disease severity in patients with HOCM. Thus, circRNAs emerge as novel biomarkers for HCM facilitating the clinical decision making in a personalized manner.

## Introduction

Hypertrophic cardiomyopathy (HCM) is one of the most commonly inherited cardiovascular diseases caused by mutations in genes encoding key cardiac sarcomeric proteins^1^. Its prevalence has been originally described with 1:500. Taking into account not only clinical manifestation but also pathogenic genetic mutations, the prevalence of HCM may increase up to 1:200, affecting as many as 20 million people worldwide recently estimated by epidemiological studies^2,3^. Only about 10 percent of patients are clinically identified, the remaining 90 percent display an unidentified cohort awaiting therapy^1^. HCM is characterized by myocardial hypertrophy and can be subdivided into (A) non-obstructive (HNCM) and (B) obstructive appearance (HOCM). Pathophysiologically, HCM is not only characterized by hypertrophy of cardiomyocytes, but also fiber disarray and progression of ventricular fibrosis. HOCM differs from HNCM clinically by the presence of a pathological increased gradient in the left ventricular outflow tract caused by the asymmetric septum hypertrophy. Such discrepancy also establishes alternative treatment regimen for these two forms of HCM. Of note, hypertrophic cardiomyopathy has various manifestations from asymptomatic status or mild clinical symptoms up to heart failure and sudden cardiac death^4^. Despite the given clinical relevance of HCM there is a lack of biomarkers that can simplify the clinical management of patients suffering from HCM.

Non-coding RNAs represent a potential class of disease-associated biomarkers investigating small non-coding RNAs such as microRNAs (miRNAs) and long non-coding RNAs (lncRNAs), respectively^5^. In the past, we and others provided evidence that miRNAs as well as lncRNAs are associated with HCM in blood and heart tissue^6–9^. In the world of RNA, circular RNAs (circRNAs) exhibit a subclass of non-coding RNAs resulting from back-splicing of exons. They are single stranded RNAs with a covalently closed circular structure and can be found nuclease-resistant in tissues as well as in fluids. The stability of circRNAs makes them ideal candidates for biomarker discovery. At the molecular level, circRNAs regulate gene expression at the transcriptional and posttranscriptional stage and are involved in multi-facetted biological processes, indeed contributing to several diseases^10,11^. Here, we identified circulating circRNAs as potential biomarkers for HCM consequently differentiating between patients with obstructive and non-obstructive hypertrophic cardiomyopathy as well as healthy subjects.

## Results

The present study included 64 patients with hypertrophic cardiomyopathy and 53 healthy control individuals. Among HCM patients there were 33 patients without and 31 with obstruction in the left ventricular outflow tract highlighted in the detailed patient characteristics (Table 1). Patients were chosen according to the diagnostic criteria based on the recent European guidelines for the diagnosis and management of hypertrophic cardiomyopathies^4^. There was no difference in the NYHA classification, numbers of syncopes, arrhythmias, positive family history and co-morbidities between HOCM and HNCM patients. At the medication level, there was no difference for HOCM and HNCM patients for beta blockers, ACE inhibitors and diuretics, but the use of AT receptor antagonists was significantly higher in HNCM patients. Comparing echocardiographic acquisition, there were no differences between left ventricular end-diastolic dimensions, size of left atrium and the thickness of the left ventricular wall between HOCM and HNCM patients. However patients with HOCM showed a significantly higher gradient in the left ventricular outflow tract due to the pathophysiology of HOCM. The incidence of mitral regurgitation was different between HOCM and HNCM group, whereby there were more minor regurgitations in the HNCM group and more medium regurgitations in the HOCM group.

**Table 1:** Patient characteristics.

Variable	Control	HCM	HCM		*P*-value	
			HNCM	HOCM	Control:HCM	HNCM:HOCM
N	53	64	33	31		
Age (years)	53.5 ± 13.7	52.6 ± 16.3	49.2 ± 15.4	56.4 ± 16.7	0.748	0.077
Male N (%)	35 (66.0)	41 (64.1)	25 (75.8)	16 (51.6)	0.848	0.068
Body mass index (kg/m^2^)		26.9 (24.4–30.4)	26.6 (24.1–30.7)	27.3 (24.5–30.0)	NA	0.619
**Echocardiogram**						
IVS (mm)		18.0 (16.0–22.0)	17.0 (14.3–22.0)	19.0 (16.0–21.0)	NA	0.205
LVEDD (mm)		43. (40.0–48.0)	43.5 (41.0–48.8)	42.0 (38.0–47–0)	NA	0.107
Aortic root (mm)		32.0 (28.0–35.0)	32.0 (27.3–35.0)	32.0 (28.8–35.3)	NA	0.702
LVPWD (mm)		12.0 (10.0–13.0)	11.0 (9.3–12.0)	12.0 (11.0–15.0)	NA	0.053
LVOT gradient (mmHg)		75.5 (12.9–115.5)	9.5 85.5–17.3)	98.5 (74.5–133.3)	NA	<0.001*
Mitral regurgitation N (%)					NA	0.002*
minor		44 (68.8)	29 (87.9)	15 (48.4)		
medium		12 (18.8)	3 (9.1)	9 (29.0)		
major		5 (7.8)	0 (0.0)	5 (16.1)		
missing		3 (4.7)	1 (3.0)	2 (6.5)		
LA size (mm)		42. (37.5–50.5)	40.0 (36.0–49.0)	44.0 (39.8–51.3)	NA	0.177
**Clinical symptoms**						
Syncope N (%)		9 (14.1)	2 (9.1)	6 (19.4)	NA	0.302
Positive family history for SCD N (%)		18 (28.1)	11 (33.3)	7 (22.6)	NA	0.408
Dyspnoea N (%)		34 (53.1)	15 (45.5)	19 (61.3)	NA	0.315
NYHA N (%)					NA	0.088
0 1		5 (7.8)	4 (12.1)	1 (3.2)		
1 2		11 (17.2)	8 (24.2)	3 (9.7)		
2 3		29 (45.3)	12 (36.4)	17 (54.8)		
3 4		15 (23.4)	5 (15.2)	10 (32.3)		
missing		4 (6.3)	4 (12.1)	0 (0.0)		
Angina pectoris N (%)		9 (14.1)	2 (6.1)	7 (22.6)	NA	0.082
Palpitations N (%)		23 (35.9)	11 (33.3)	12 (38.7)	NA	0.797
Peripheral edema N (%)		9 (14.1)	6 (18.2)	3 (9.7)	NA	0.474
Arrhythmias		31 (48.4)	19 (57.6)	12 (38.7)	NA	0.204
Atrial Fibrillaton		6 (9.4)	4 (12.1)	2 (6.5)	NA	0.672
Mitral valve murmur		26 (40.6)	7 (21.2)	19 (61.3)	NA	0.004*
**Co-morbidities**						
Hypertension N (%)		31 (48.4)	17 (51.5)	14 (45.2)	NA	0.612
Diabetes mellitus N (%)		6 (9.4)	3 (9.1)	3 (9.7)	NA	1.000
Coronary artery disease N (%)		13 (20.3)	5 (15.2)	8 (25.8)	NA	0.534
Myocardial infarction		4 (6.3)	2 (6.1)	2 (6.5)	NA	1.000
COPD		4 (96.9)	0 (0.0)	4 (12.9)	NA	0.113
**Drugs**						
Beta blockers N (%)		50 (78.1)	25 (72.7)	26 (83.9)	NA	0.536
ACE inhibitors		19 (29.7)	10 (30.3)	9 (29.0)	NA	1.000
AT1 inhibitors		10 (15.6)	9 (27.3)	1 (3.2)	NA	0.013*
Diuretics		23 (35.9)	1 (97.0)	13 (41.9)	NA	0.439
Calcium antagonists N (%)		18 (28.1)	8 (24.2)	10 (32.3)	NA	0.585
Anticoagulation drugs N (%)		32 (50.0)	15 (45.5)	17 (54.8)	NA	0.617

Data are presented as frequencies (percentages) for categorical variables. Continuous variables with normal distribution are presented as mean ± standard deviation. Continuous parameters with skewed distributions as median (interquartile range). *Statistically significant. NA: Not applicable.

Data are presented as frequencies (percentage) for categorical variables. Continuous variables with normal distribution are presented as mean ± standard deviation. Continuous parameters with skewed distributions as median (interquartile range)^32^. HCM: hypertrophic cardiomyopathy; HNCM: non-obstructive hypertrophic cardiomyopathy; HOCM: hypertrophic obstructive cardiomyopathy; NA: Not applicable.

IVS = interventicular septum size; LVEDD = left ventricular end-diastolic diameter; LVPWD = left ventricular posterior wall thickness end diastole; LVOT gradient = left ventricular outflow tract gradient maximum; LA = left atrium; AT1 = angiotensin II receptor antagonist; ACE = angiotensin-converting enzyme; COPD = chronic obstructive pulmonary disease; SCD = sudden cardiac death.

**Figure 1:**
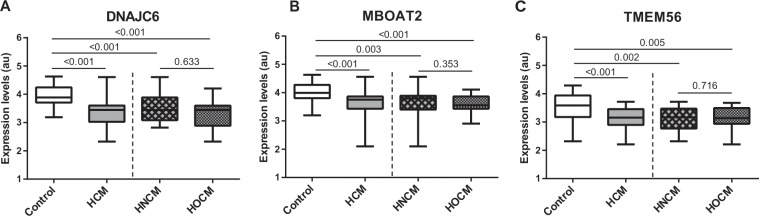
Expression levels of circular RNAs were measured in serum of patients in the study groups: (**A**) circDNAJC6; (**B**) circMBOAT2; (**C**) circTEM56. Differences between groups were analyzed using Kruskal–Wallis test and/or Mann–Whitney U test. Data represent the medians (IQR), minimum and maximum. P‐values describe the significance level of differences for each comparison. HCM: hypertrophic cardiomyopathy; HNCM: non-obstructive hypertrophic cardiomyopathy; HOCM: obstructive hypertrophic cardiomyopathy.

**Figure 2:**
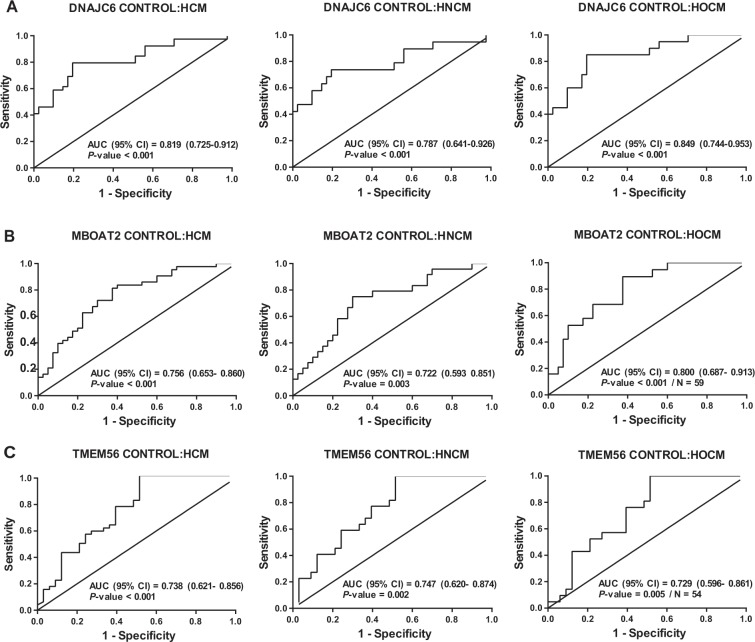
ROC curve analyses of circulating circular RNAs. (**A**) circDNAJC6; (**B**) circMBOAT2; (**C**) circTMEM56. Data are presented as the area under the ROC curve (AUC) and 95% confidence intervals (CI). HCM: hypertrophic cardiomyopathy; HNCM: non-obstructive hypertrophic cardiomyopathy; HOCM: obstructive hypertrophic cardiomyopathy.

In line to previously published data we investigated the concentration of several circRNAs known to be abundant in peripheral blood^12,13^ and were able to detect 3 circRNAs (circDNAJC6, circMBOAT2 and circTMEM56) in serum via quantitative Real-time PCR (detailed information about circRNAs isoforms are given in Supplemental Table S1). The expression levels of circRNAs DNAJC6, MBOAT2 and TMEM56 were significantly higher in control subjects than in patients with HCM (Fig. 1A–C). The inverse association between circRNA levels and the presence of HCM remained unchanged even after adjusting for age and sex (Table 2). Analyzing both groups HOCM and HNCM revealed similar levels of the circRNA set (Fig. 1A–C). To further underline these results, we performed an additional ROC curve analysis indicating that all circRNAs, separately or in combination, demonstrated a strong discrimination value when comparing control subjects with HCM, HNCM or HOCM patients [area under the ROC curve (AUC) from 0.722 to 0.949] **(**Fig. 2A–C and Suppl. Fig. S2A–C). As expected, circRNAs showed a low AUC for the comparison between HOCM and HNCM (AUC from 0.532 to 0.583) (Suppl. Fig. S1A–C). Nonetheless, the discrimination for HNCM vs HOCM was improved when combining the three circRNAs (AUC = 0.650) (Suppl. Fig. S2D).

**Table 2:** Association between circulating circRNAs and HCM.

	Model 1	*P*-value	Model 2	*P*-value
	OR (95% IC)		OR (95% IC)	
DNAJC6	0.048 (0.012–0.198)	<0.001*	0.033 (0.007–0.164)	<0.001*
MBOAT2	0.074 (0.017–0.317)	<0.001*	0.070 (0.016–0.309)	<0.001*
TMEM56	0.135 (0.041–0.447)	0.001*	0.134 (0.040–0.447)	0.001*

Model 1: Unadjusted; Model 2: Adjusted for age and sex. OR: Odd ratio, 95% CI 95% confidence interval. *: Statistically significant.

Association between circulating circular RNAs (circRNAs) in control group and hypertrophic cardiomyopathy group (Model 1 and 2). Expression level of circRNAs were additionally adjusted for age and sex in Model 2. Model 1: unadjusted; Model 2: adjusted for age and sex. OR = odds ratio, 95% CI = 95% confidence interval.

**Figure 3:**
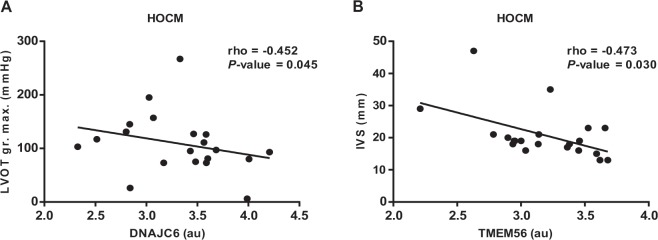
Correlations between circulating circular RNAs and echocardiographic parameters in patients with obstructive hypertrophic cardiomyopathy (HOCM). (**A**) circDNAJC6 vs LVOT gr. Max.; (**B**) circTEME56 vs IVS. Correlations between variables were analyzed using Spearman’s rho coefficient. LVOT gr. Max.: maximum gradient in left ventricular outflow tract. IVS: interventricular septum.

We also performed correlation analyses to explore the potential association of HCM with different echocardiographic parameters that are the main characteristics for HOCM. As shown in Fig. 3, we observed a direct significant correlation between circRNAs circDNAJC6 and circTMEM56 and left ventricular outflow tract gradient (LVOT gr.max.) as well as thickness of interventicular septum (IVS) in this patient group. No correlations were observed between circRNAs and echocardiographic parameters in the whole HCM population or HNCM subgroup (data not shown). Taken together, the expression level of circRNAs is negatively correlated with LVOT gradient and IVS thickness in patients with HOCM indicating the severity of this obstructive cardiac disease and helping to improve early discovery.

## Discussion

Herein, we quantitatively explored for the first time circulating circRNAs in patients with HCM. The major finding of our study is that abundancy of circulating circRNAs circTMEM56, circDNAJC6 and circMBOAT2 is significantly lower in patients with HCM than in healthy controls. Importantly, the three circRNAs show a good performance as biomarkers of HCM, as suggested by their discrimination value. In addition, the level of circRNAs circTMEM56 and circDNAJC6 is negatively correlated to the severity of left ventricular obstruction and thickness of interventricular septum in HOCM group. A separate classification between HNCM and HOCM can only be made by a combinatorial approach, as the individual circRNAs do not show a significant difference between these subgroups of HCM.

Noteworthy, circRNAs have emerged as an interesting class of biomarkers being highly conserved and resistant to cellular influences of RNAses^14^. Several global profiling studies identified and characterized multiple circRNAs in different tissues as well as in peripheral blood^12,13,15^. circRNAs can act on gene expression in different ways, e.g. functioning as miRNA-sponges or regulating transcription^16–18^. On the opposite, circRNAs can be regulated by RNA binding proteins like Rbm20 in dilated cardiomyopathy^19^ or Quaking in doxorubicin-induced cardiomyopathy^20^. There is growing evidence that circRNAs can be secreted from diseased tissue into the extracellular space via exosomes^13,21^. Iparraguirre *et al*. examined the role of circulating circRNAs in autoimmune diseases and reported that circRNA ANXA2 from peripheral blood can be used as a biomarker for multiple sclerosis^12^. Endogenous circRNAs are also implicated in cancer development and can serve as biomarkers. In non- small cell lung cancer a circRNA derived from exons of the FARSA gene and called circFARSA, was observed to be enriched in cancerous tissues. Next to that circFARSA was more abundant in patients’ plasma than controls, therefore demonstrating evidence of its role as a potential noninvasive biomarker for malignancy of non-small cell lung cancer^11^. Switching to the field of cardiovascular diseases, circRNA CDR1as is upregulated in hypoxic cardiomyocytes and in myocardial infarction in mice^22^. Another circRNA MICRA was shown to be less abundant in peripheral blood of patients with myocardial infarction and in line low levels of MICRA are associated with the higher risk of left ventricular dysfunction^23,24^. The circRNA molecule circFOXO3 is involved in doxorubicin-induced senescence and promotes cardiac senescence by modulating cytoprotective proteins in different cellular compartments^25^. Experimental evidence exists that circRNAs are also implicated in cardiac fibrosis, e.g. circRNA_000203 and circRNA_010567^26,27^ both leading to increased levels of pro-fibrotic proteins by sponging corresponding miRNAs. At the clinical stage focusing on dilated cardiomyopathy (DCM) mutations in RNA binding protein Rbm20 are associated with loss of circRNAs from titin gene and result in the development of dilative phenotype^19^. Siede *et al*. have recently shown that several circRNAs, e.g. circDNAJC6, can be dynamically expressed in dilated hearts and human induced pluripotent stem cell derived cardiomyocytes subjected to disease development and stress response^28^. In contrast, little is known about circRNAs in HCM. Following up the observation of decreased circDNAJC6 levels in bioptic material from patients with DCM^28^, we examined the expression level of this circRNA in serum of patients with HCM. Our data indicate that DNAJC6 expression was lower in HMC group compared to controls and negatively correlates with the maximum gradient in LVOT. We examined the correlation of more circRNAs with HCM characteristics determined via echocardiography. While there was no correlation in the HNCM group between circRNAs and echocardiographic parameters, circTMEM56 and circDNAJC6 significantly negatively correlated with echocardiographic parameters characteristic for HOCM (LVOT grad. max and IVS). Therefore, HOCM patients with the lowest circRNA expression level had significantly higher LVOT grad. max. and IVS. Thus, both circRNAs circTMEM56 and circDNAJC6 may serve as an indicator of disease severity in patients with HOCM. Further studies now should investigate the importance of the interaction between circRNAs and clinical severity of HOCM in order to facilitate therapeutic decisions, e.g. implantation of cardioverter/defibrillator (ICD) or myocardial reduction therapy (TASH or myectomy).

According to the current clinical guidelines genetic testing of patients is recommended as a class I indication in the presence of symptoms and signs of disease to confirm the diagnosis^4^. This may contribute to facilitate clinical risk stratification^29^. Due to the still limited therapeutic consequences of genetic testing and according to our clinical experience the willingness of patients to perform genetic testing is low. We thus cannot supply genetic association information of cardiomyopathy and circRNA abundancy and extension of genetic analysis is a major goal for upcoming studies.

The strength of the present study is the fact that we not only compared healthy subjects with HCM patients in general, but also differentiate between HNCM and HOCM to further identify a correlation between circRNAs and disease severity. Nevertheless, results of this medium-size study needs to be replicated by larger and independent study cohorts.

In conclusion, this is the first study indicating the abundancy of disease-relevant circulating circRNAs in HCM and highlighting the importance of a set of circRNAs as possible novel indicators of HCM.

## Methods

### Patient data

Patients with HNCM/HOCM were enrolled at the Special Outpatient Clinic for HCM, Department of Cardiology and Angiology (Hannover Medical School). We obtained written informed consent from all patients and the study was approved by the local ethical committee of Hannover Medical School.

The diagnosis of HCM was based on the recent European guidelines for the diagnosis and management of hypertrophic cardiomyopathies^4^ and mainly included presence of a hypertrophic cardiac septum (≥15 mm) or combined presence of a hypertrophic cardiac septum (≥13 mm) and positive family history and/or ECG abnormalities. To differentiate between non-obstructive and obstructive subtype, HOCM was defined by a left ventricular outflow tract gradient ≥ 30 mmHg. All methods were carried out in accordance with relevant guidelines and regulations.

Blood from healthy do nors (controls) was obtained from the Hannover Medical School blood donation service.

### RNA isolation from patient serum

As described previously^6,7,30^, collected serum blood samples were centrifuged at 2000 × g for 10 minutes at room temperature. After separation of corpuscular components, the liquid supernatant was stored at −80 °C in RNase/DNase free tubes. RNA was isolated with the miRNeasy Mini Kit (Qiagen) according to the manual. For normalization Caenorhabditis elegans miR-39-3p was added as a spike-in RNA during the process^6,7,30^.

### Reverse transcription and real-time PCR of circRNAs

Isolated RNA was transcribed to complementary DNA (cDNA) using cDNA Synthesis Kit (Biozym) according to manufacturer’s manuals and as described previously^6,7^. TaqMan MircoRNA Reverse Transcription Kit (Applied Biosystems) was applied to synthesize cDNA of cel-miR-39-3p working as control. According to the literature^12,13^ specific primers for circ_0005402, circ_0035560, ANXA2, MBOAT2, TMEM56 and DNAJC6 were validated and afterwards quantitative real-time PCR was performed amplifying the cDNA of the four last mentioned circRNAs using iQSYBR Green Supermix (Bio-Rad). For amplifying cel-miR-39 with quantitative real-time PCR, a specific TagMan MicroRNA assay and ViiA7 machine (Applied Biosystems) was used.

Following primers were used in this study:

**Table:** 

circ_0005402 forward	TTTCGGACACATCTGGTGAC
circ_0035560 forward	CACCTGGAGACGGTGATTTT
rev-circ_universal	CCGCTCAGCATCAAAGTTAGT
MBOAT2 forward	AGTGCAAGATAAAGGCCCAAA
MBOAT2 reverse	TGATCATCATAGGAGTGGAGAACA
TMEM56 forward	CATCATTGTGCGTCCCTGTATG
TMEM56 reverse	GCTGAGACTATTGAAACCTGGAGA
DNAJC6 forward	CCAGACATCTTGACCACTACACA
DNAJC6 reverse	ATGTGTCTTTGAGGGTGTCTTT

### Statistical analysis

Comprehensive statistical evaluation was performed as described previously^31,32^.

In specific we used the statistical software package R (www.r-project.org) for statistical analyses. Descriptive statistics were used to summarize the characteristics of the study population. Kolmogorov-Smirnov test was used to test normality. Data were presented as the means ± standard deviation (SD) for continuous variables with normal distributions, medians (interquartile range) for continuous variables with skewed distributions and as frequencies (percentage) for categorical variables. Continuous variables were compared between groups using Student´s t test, Krukal–Wallis test or Mann–Whitney U test. Categorical variables were compared between groups using Fisher’s exact test or *chi*-*squared test*. Spearman’s rho coefficient was used to assess the correlation between continuous variables. Logistic regression analyses were performed to examine in detail the association between circRNAs and HCM, HNCM or HOCM. To establish whether the observed association could be influenced by potential cofounding factors, the models were adjusted by age and sex. The results were presented as odds ratio (OR) and 95% confidence intervals (CI). Receiver operating characteristic (ROC) curves were constructed for circRNAs using the area under the ROC curve (AUC) as the global discrimination value measure. P-value < 0.05 was considered statistically significant.

## Supplementary information

Supplementary Information.

## Data Availability

All data generated or analyzed during this study are included in this published article and its Supplementary Information files.
