# Supplementary material for: Serum circular RNAs act as blood-based biomarkers for hypertrophic obstructive cardiomyopathy
Source: Sci Rep. 2019 Dec 30;9:20350. doi: 10.1038/s41598-019-56617-2 (PMC6937321; doi:10.1038/s41598-019-56617-2)
Supplement: Supplementary file 1 — Supplementary Information. [file 41598_2019_56617_MOESM1_ESM.pdf]

SUPPLEMENTARY INFORMATION

Serum circular RNAs act as blood-based biomarkers for hypertrophic obstructive cardiomyopathy

Kristina Sonnenschein, MD[1,2] *, Adriana Luisa Wilczek[1] *, David de Gonzalo-Calvo, PhD[1,3,4,5] Angelika Pfanne[1], Carolin Zwadlo[2], Udo Bavendiek[2], Johann Bauersachs, MD[2,7], Jan Fiedler, PhD[1]*,Thomas Thum, MD, PhD[1,6,7]*

[1] Institute of Molecular and Translational Therapeutic Strategies (IMTTS), Hannover Medical School, Hannover, Germany

[2] Department of Cardiology and Angiology, Hannover Medical School, Hannover, Germany

[3] Institute of Biomedical Research of Barcelona (IIBB) - Spanish National Research Council (CSIC), 08036, Barcelona, Spain.

[4] CIBERCV, Institute of Health Carlos III, 28029, Madrid, Spain.

[5] Biomedical Research Institute Sant Pau (IIB Sant Pau), 08025, Barcelona, Spain.

[6] National Heart and Lung Institute, Imperial College London, London, UK

[7] Excellence Cluster REBIRTH, Hannover Medical School, Hannover, Germany

Supplemental Table S1: In silico analysis of circRNA isoforms.

position (genome browser link)	strand	circRNA ID	genomic length	spliced length	best transript	gene symbol	circRNA study
chr1:65858293-65858516	-	hsa_circ_0113915	223	223	NM_014787	DNAJC6	Rybak2015
chr1:65830317-65831879	+	hsa_circ_0002454	1562	350	NM_014787	DNAJC6	Jeck2013, Maass2017, Rybak2015, Salzman2013
chr1:65830317-65860715	+	hsa_circ_0113910	30398	1845	NM_014787	DNAJC6	Rybak2015
chr1:65830317-65864564	+	hsa_circ_0012885	34247	1914	NM_014787	DNAJC6	Salzman2013
chr1:65871552-65874466	+	hsa_circ_0113916	2914	407	NM_014787	DNAJC6	Rybak2015
chr1:65830317-65855310	+	hsa_circ_0012884	24993	1275	NM_014787	DNAJC6	Salzman2013
chr1:65830317-65845207	+	hsa_circ_0012883	14890	473	NM_014787	DNAJC6	Rybak2015, Salzman2013
chr1:65831730-65831879	+	hsa_circ_0012886	149	149	NM_014787	DNAJC6	Salzman2013
chr1:65858293-65858394	-	hsa_circ_0113914	101	101	NM_014787	DNAJC6	Rybak2015
chr1:65830317-65871816	+	hsa_circ_0113911	41499	2298	NM_014787	DNAJC6	Rybak2015
chr1:65811293-65831879	+	hsa_circ_0113909	20586	463	ENST00000263441.7	DNAJC6	Rybak2015
chr1:65849875-65855310	+	hsa_circ_0113912	5435	802	NM_014787	DNAJC6	Rybak2015
chr1:65854018-65855310	+	hsa_circ_0113913	1292	355	NM_014787	DNAJC6	Rybak2015
chr2:9083315-9122273	-	hsa_circ_0121179	38958	23726	ENST00000305997.3	MBOAT2	Rybak2015
chr2:9000742-9008679	-	hsa_circ_0121168	7937	454	ENST00000305997.3	MBOAT2	Rybak2015
chr2:9048750-9114564	-	hsa_circ_0121171	65814	16113	ENST00000305997.3	MBOAT2	Rybak2015
chr2:9028147-9098771	-	hsa_circ_0008847	70624	376	NM_138799	MBOAT2	Jeck2013, Maass2017, Rybak2015, Salzman2013
chr2:9048750-9114564	-	hsa_circ_0141916	65814	16113	ENST00000305997.3	MBOAT2	Maass2017
chr2:9079949-9099994	-	hsa_circ_0121175	20045	4813	ENST00000305997.3	MBOAT2	Rybak2015
chr2:9083315-9083393	-	hsa_circ_0052511	78	78	NM_138799	MBOAT2	Salzman2013
chr2:9079949-9098771	-	hsa_circ_0000973	18822	3590	NM_138799	MBOAT2	Jeck2013, Maass2017, Memczak2013, Rybak2015
chr2:9083315-9127890	-	hsa_circ_0141918	44575	29343	ENST00000305997.3	MBOAT2	Maass2017

chr2:9000742-9008679	-	hsa_circ_0141915	7937	454	ENST00000305997.3	MBOAT2		Maass2017
chr2:9083315-9102747	-	hsa_circ_0121177	19432	4200	ENST00000305997.3	MBOAT2		Rybak2015
chr2:9017159-9048846	-	hsa_circ_0052507	31687	391	NM_138799	MBOAT2		Rybak2015, Salzman2013
chr2:9048750-9127890	-	hsa_circ_0121173	79140	29439	ENST00000305997.3	MBOAT2		Rybak2015
chr2:9048750-9048846	-	hsa_circ_0052509	96	96	NM_138799	MBOAT2		Salzman2013
chr2:9022640-9098771	-	hsa_circ_0005079	76131	431	NM_138799	MBOAT2		Jeck2013, Maass2017, Rybak2015, Salzman2013
chr2:9017116-9098771	-	hsa_circ_0121169	81655	658	ENST00000305997.3	MBOAT2		Rybak2015
chr2:9013237-9098771	-	hsa_circ_0052506	85534	808	NM_138799	MBOAT2		Rybak2015, Salzman2013
chr2:9008575-9028203	-	hsa_circ_0052504	19628	592	NM_138799	MBOAT2		Rybak2015, Salzman2013
chr2:9017159-9098771	-	hsa_circ_0052508	81612	615	NM_138799	MBOAT2		Maass2017, Rybak2015, Salzman2013
chr2:8996700-9098771	-	hsa_circ_0052501	102071	3596	NM_138799	MBOAT2		Salzman2013
chr2:9019778-9020143	-	hsa_circ_0000971	365	365	NM_138799	MBOAT2		Memczak2013
chr2:9022640-9083393	-	hsa_circ_0121170	60753	285	ENST00000305997.3	MBOAT2		Rybak2015
chr2:9083315-9114564	-	hsa_circ_0141917	31249	16017	ENST00000305997.3	MBOAT2		Maass2017
chr2:9083315-9098771	-	hsa_circ_0007334	15456	224	NM_138799	MBOAT2		Jeck2013, Maass2017, Rybak2015, Salzman2013
chr2:9002400-9098771	-	hsa_circ_0052503	96371	1429	NM_138799	MBOAT2		Salzman2013
chr2:9000742-9098771	-	hsa_circ_0052502	98029	1262	NM_138799	MBOAT2		Rybak2015, Salzman2013
chr2:9083315-9127890	-	hsa_circ_0121180	44575	29343	ENST00000305997.3	MBOAT2		Rybak2015
chr2:9079949-9083393	-	hsa_circ_0121174	3444	3444	ENST00000305997.3	MBOAT2		Rybak2015
chr2:9048750-9122273	-	hsa_circ_0121172	73523	23822	ENST00000305997.3	MBOAT2		Rybak2015
chr2:9083315-9143876	-	hsa_circ_0052512	60561	432	NM_138799	MBOAT2		Salzman2013
chr2:9083315-9114564	-	hsa_circ_0121178	31249	16017	ENST00000305997.3	MBOAT2		Rybak2015
chr2:9048750-9098771	-	hsa_circ_0000972	50021	320	NM_138799	MBOAT2		Jeck2013, Maass2017, Memczak2013,

							Rybak2015, Salzman2013
chr2:9098625-9098771	-	hsa_circ_0052513	146	146	NM_138799	MBOAT2	Salzman2013
chr2:9079949-9114564	-	hsa_circ_0121176	34615	19383	ENST00000305997.3	MBOAT2	Rybak2015
chr2:9008575-9098771	-	hsa_circ_0052505	90196	912	NM_138799	MBOAT2	Rybak2015, Salzman2013
chr2:9083315-9137472	-	hsa_circ_0121181	54157	38925	ENST00000305997.3	MBOAT2	Rybak2015
chr2:9048750-9083393	-	hsa_circ_0052510	34643	174	NM_138799	MBOAT2	Rybak2015, Salzman2013
chr1:95609446-95639445	+	hsa_circ_0005720	29999	484	NM_001199679	TMEM56	Jeck2013, Maass2017, Rybak2015, Salzman2013
chr1:95616880-95663161	+	hsa_circ_0013248	46281	6225	NM_001199679	TMEM56	Salzman2013
chr1:95616880-95616975	+	hsa_circ_0013247	95	95	NM_001199679	TMEM56	Salzman2013
chr1:95657105-95663161	+	hsa_circ_0013249	6056	6056	NM_001199679	TMEM56	Salzman2013
chr1:95609446-95644494	+	hsa_circ_0114564	35048	5533	NM_001199679	TMEM56	Rybak2015
chr1:95609446-95696550	+	hsa_circ_0114565	87104	577	ENST00000604534.1	TMEM56	Rybak2015
chr1:95609446-95616975	+	hsa_circ_0000095	7529	410	NM_001199691	TMEM56-RWDD3	Maass2017, Memczak2013, Rybak2015, Salzman2013

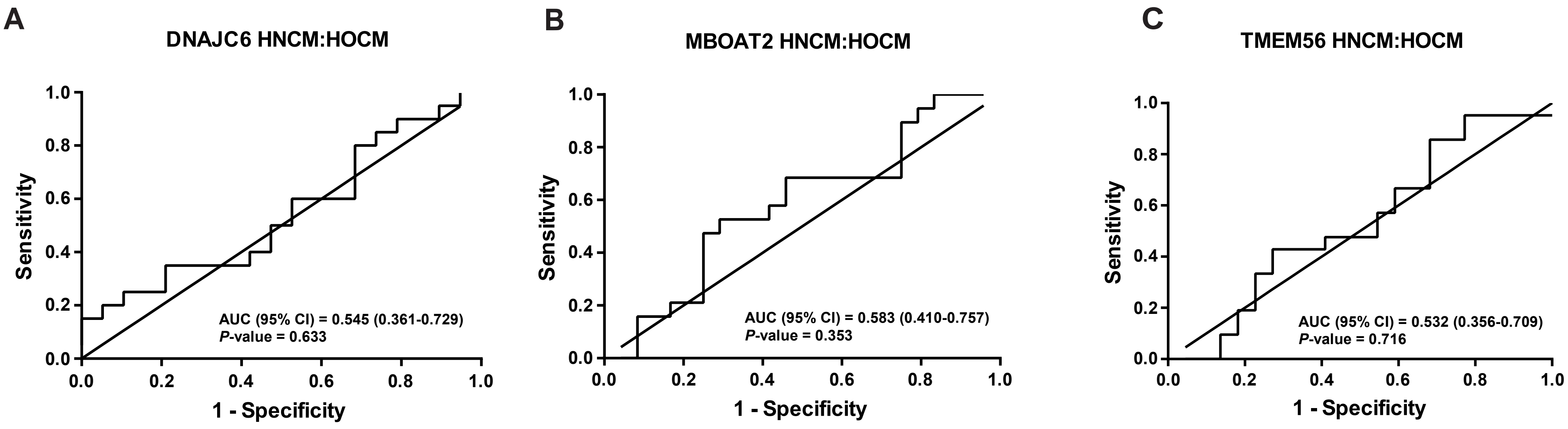

Supplemental Figure S1. ROC curve analyses of circulating circular RNAs (HNCM vs HOCM). **A)** circDNAJC6; **B)** circMBOAT2; **C)** circTMEM56. Data are presented as the area under the ROC curve (AUC) and 95% confidence intervals (CI). HNCM: non-obstructive hypertrophic cardimyopathy. HOCM: obstructive hypertrophic cardiomyopathy.

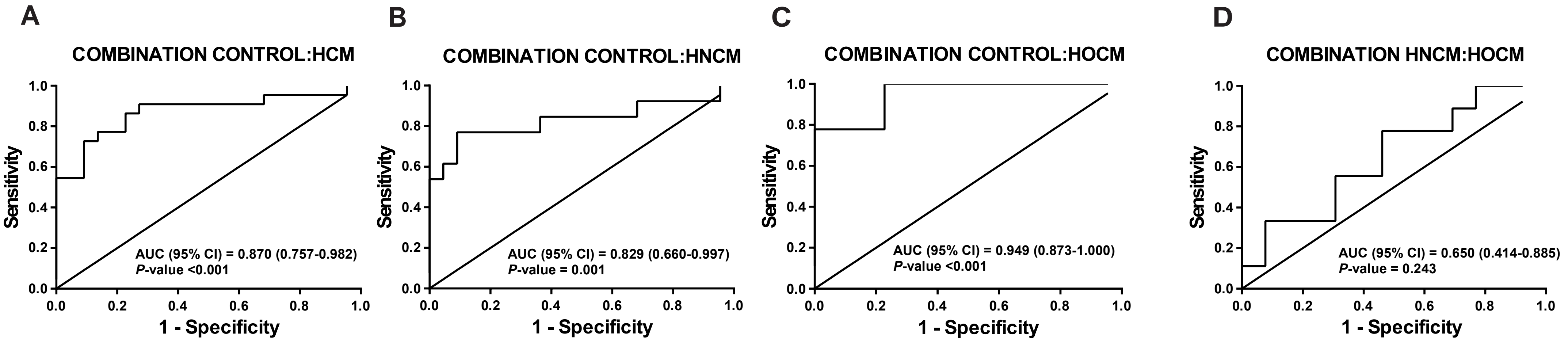

Supplemental Figure S2. ROC curve analyses of the combination of all circulating circular RNAs. **A)** control group vs hypertrophic cardiomyopathy (HCM) group; **B)** control group vs non-obstructive hypertrophic cardiomyopathy (HNCM) group; **C)** control group vs obstructive hypertrophic cardiomyopathy (HOCM) group; D) HNCM group vs HOCM group. Data are presented as the area under the ROC curve (AUC) and 95% confidence intervals (CI).
